# Editorial

**Published:** 1842-01-22

**Authors:** 


### THE MEDICAL EXAMINER.

PHILADELPHIA, JANUARY 22, 1842.

Dr. H. L .Byard, of Salem, Sumpter district, South Carolina, has furnished us with a sketch of the prevailing form of fever met with in his practice; it appears that it was remittent, with more than the usual complications of gastritis. Whether this form prevailed throughout the Southern States, or was limited to a particular district of South Carolina, we do not know. The epidemic tendency of the season appears to have been rather of the congestive than inflammatory type.

